# Evaluation of Platelet-Rich Plasma and Neutrophil Antimicrobial Extract as Two Autologous Blood-Derived Agents

**DOI:** 10.1007/s13770-017-0035-4

**Published:** 2017-05-19

**Authors:** Tomasz Szponder, Joanna Wessely-Szponder, Anna Smolira

**Affiliations:** 1 Department and Clinic of Animal Surgery, Faculty of Veterinary Medicine, University of Life Sciences, Głęboka 30, 20-612 Lublin, Poland; 2 Department of Pathophysiology, Chair of Preclinical Veterinary Sciences, Faculty of Veterinary Medicine, Akademicka 12, 20-033 Lublin, Poland; 3 Mass Spectrometry Laboratory Institute of Physics, Maria Curie Sklodowska University, Pl. M. Curie-Skłodowskiej 1, 20-031 Lublin, Poland

**Keywords:** Antimicrobial peptides neutrophil extract, Cathelicidin, Platelet-rich plasma

## Abstract

The platelet-rich plasma (PRP) and antimicrobial peptides neutrophil extract (AMP extract) were prepared from rabbit neutrophils as two autologous blood-derived preparations, which could be applied locally to enhance healing process of tissues. Both preparations were analyzed using the MALDI TOF method for accurate qualitative assay. Growth factors (PDGF and VEGF) and microbicidal protein were reported in PRP. In AMP extract α-defensins, namely; NP-1, -2, -3a, -3b, -4, and -5 and cathelicidins represented among other by 15-kDa antibacterial protein (p15s) were detected. In the second part of our study the influence of antimicrobial extract on macrophages *in vitro* was tested. Then, degranulation of neutrophils *in vitro* and generation of reactive intermediates by these cells under the influence of AMP extract were assessed. As estimated, the addition of AMP extract into cultures of macrophages decreased superoxide anion generation after 5 days of incubation. Furthermore, AMP extract inhibited degranulation and respiratory burst in neutrophils, therefore in this regard it suppress proinflammatory effect of two studied populations of leukocytes.

## Introduction

Some naturally occurring substances, so called “autologous biomaterials” have a potential for enhancing the bone and soft tissue healing and help fight infection [1]. One of these biomaterials is the platelet-rich plasma (PRP), as a preparation of concentrated autologous platelets containing growth factors and other components such as the antimicrobial agent PMP (platelet microbicidal proteins) [2–4].

Some antimicrobial peptides i.e. defensins and cathelicidins are present in neutrophils, which are first leukocytes that rapidly and efficiently reach the site of inflammation and injury [5]. Antimicrobial peptides are not only microbicidal molecules but also exert many regulatory functions during inflammation, they can act as signalling molecules with potential for modulation of wound repair and inflammation by influence on inflammatory cells [5–7]. Among these cells, macrophages (Mfs) initially contribute to the elimination of pathogens and the elicitation of an inflammatory reaction (as M1). When the infection is resolved their function may shift towards resolution of inflammation and tissue repair (as M2). Thus, the polarization phenotype adopted by a macrophage can have a major influence on healing process, and antimicrobial peptides i.e. defensins and cathelicidins may participate in this regulation [8].

Neutrophil-derived antimicrobial peptides were applied previously, especially in treatment of osteomyelitis [9, 10]. However, combined application of both preparations, namely, neutrophil and platelet crude extract, was suggested by Anderson et al. [11], but not used previously. Because both autologous preparations might be applied locally in surgical and orthopedic conditions to enhance healing, our first aim was to prepare PRP with limited amount of WBC, and to prepare the antimicrobial peptides (AMP) neutrophil extract containing natural antimicrobial peptides. In the second part of our study we evaluated the influence of this AMP extract on activity of neutrophils and Mfs *in vitro*.

## Materials and methods

### Animals and study design

The study was carried out on 6 healthy New Zealand White (NZW) rabbits, males with the body weight between 3500 and 4000 g. The rabbits were individually caged, fed with a standard balanced rabbit chow and provided with water *ad libitum*. All animals were treated according to the guidelines for laboratory animal treatment and care. The study protocol was approved by the Local Ethics Committee of University of Life Sciences in Lublin (Decision no. 12/2014) and experiment was performed in accordance with animal protection regulations.

### Preparation of PRP

An 8.5 ml sample of blood was collected from the each animal through the marginal auricular vein into the monovette containing citrate phosphate, dextrose and adenosine (CPDA) (PRP Kit Curasan AG). A two-step centrifugation was applied. First, the blood samples were centrifuged at 160×*g* for 20 min at room temperature to separate the plasma containing the platelets (PLT) from red blood cells. The plasma fraction was collected and centrifuged (400×*g*, 15 min). The top layer (platelet poor plasma—PPP) was removed, the remaining volume was platelet rich plasma (PRP) [1, 4].

The complete blood cells count analysis was performed in the whole blood before plateletpheresis, in platelet-rich plasma PRP, and platelet poor plasma PPP using the Vet EXIGO analyser (Boule Medical AB). The % yield was calculated as following: (number of platelets in PRP/number of platelets in the whole blood) × 100. Before experiment a preliminary study was performed to validate platelet counts on Vet EXIGO analyser with samples counted manually with a hemocytometer.

### Preparation of the neutrophil crude extract of AMP

For isolation of neutrophils the same rabbits were used. Intervals between blood sampling were at least two weeks. Blood (2–3 ml) was collected from the marginal auricular vein into the syringe with an anticoagulant 3.8% citrate. After the red blood cells were lysed by the addition of 0.83% ammonium chloride at the ratio of 3:1 to the obtained blood sample, the remaining pellet was washed twice with phosphate-buffered saline (PBS-Biomed, Lublin, Poland). The final cells (>85% of PMN on the May-Grunwald-Giemsa-stained preparations) were then homogenized to release the neutrophil granules. These granules were collected (25,000×*g*, 40 min, 4 °C), suspended in 10% acetic acid and stirred overnight at 4 °C to extract the antimicrobial peptides. The solution containing the peptides was separated from the granules (25,000×*g*, 20 min, 4 °C) and obtained extract was considered as AMP neutrophil extract. The portions of this extract were lyophilized and stored at −20 °C for further analysis [7]. The portions of 40 µg/ml of AMP extract were dissolved in PBS and used for stimulation of cultures of macrophages or neutrophils.

### Analysis of autologous blood products by MALDI TOF MS

Before MALDI TOF analysis the gel filtration chromatography using the Sephadex G-50 (Fine, Sigma-Aldrich) column was made for separation of components of obtained AMP extract according to their molecular weights [7, 12]. The fraction 1 (MW from 2500 to 17,500 Da) and the fraction 2 (MW from 2750 to 4750 Da) were collected, lyophilised, and stored at −20 °C for further analysis.

The mass analysis of the components of PRP was carried out by the MALDI method with sinapinic acid (3,5–dimethoxy-4-hydroxycinnamic acid, M = 224.2 Da, Sigma-Aldrich, Poland) used as a matrix, whereas antimicrobial peptides from the neutrophil crude extract were analyzed using the CCA matrix (α-cyano-4-hydroxycinnamic acid 189.2 Da, Sigma-Aldrich, Poland) [13].

### Monocytes isolation and generation of monocytes-derived macrophages

Few weeks after previous blood drawing, mononuclear cells were isolated from about 5 ml of heparinized rabbit blood using Lymphoprep density gradient centrifugation. After being counted, cells were plated at a density of 1.0 × 10^6^ cells/ml and cultured at 37 °C and 5% CO_2_ for 24 h in Dulbecco’s Modified Eagle’s Medium (DMEM) medium with 10% calf serum. The adherent cells were cultured for additional 48 h to allow monocytes to mature to functional macrophages. Then, the cell suspensions were supplemented as follows: the control group (DMEM) was supplemented with PBS, the LPS group with LPS from E. coli serotype 055:B5 (Sigma-Aldrich, Poland) at concentration of 1 μg/ml, the AMP group with 40 µg/ml of AMP extract reconstructed from lyophilizate in PBS, the DEX group with Dexamethasone at concentration of 100 nM. Then, the samples were incubated for 24 h at 37 °C and 5% CO_2_. Thereafter, the cultures were subjected morphological and functional analysis, which was repeated after 3 and 5 days of incubation [8].

### Functional and morphological characterization of cultured macrophages

Nitric oxide level was determined by Griess reaction described previously [12]. The obtained values were expressed as a concentration of nitrite, as the stable product of NO, which accumulates in the medium and were calculated from a NaNO_2_ standard curve.

Superoxide production was measured using the method described previously [12]. In brief, the medium from cultures of macrophages with different stimulators were incubated with 0.1% nitroblue tetrazolium (NBT-Sigma-Aldrich, Poland) solution at room temperature for 15 min and then absorbance was read. All tests were done in duplicate.

Every subsequent days of culture macrophages were subjected to microscopic analysis of the morphology.

### Isolation and stimulation of rabbit neutrophils

Rabbit neutrophils were isolated from peripheral blood as described in the chapter 2.3. The cell suspensions of purified neutrophils were adjusted to a final concentration of 2 × 10^6^ cells/ml and then were supplemented as follows: the control group (PBS) with PBS, the LPS group (LPS) with LPS from *E. coli* serotype 055:B5 (Sigma-Aldrich, Poland) in concentration of 1 µg/ml for stimulating proinflammatory conditions, the LPS/AMP group (AMP) was stimulated with 1 µg/ml of LPS, the DEX group with DEX at concentration of 100 nM for stimulation of antiinfammatory conditions. Then, cultures were incubated for 30 min at 37 °C and 5% CO_2_. Thereafter, the AMP group was stimulated with extract of AMP at concentration of 40 µg/ml dissolved in PBS, other cultures were supplemented with equal volumes of PBS at the same time. Next, the cultures were incubated for 60 min at 37 °C and 5% CO_2_ and functional analyses were conducted. Then, the incubation was continued up to 24 h at 37 °C and 5% CO_2_ and all assays were repeated [12].

### Assay of the activity of rabbit neutrophils

Neutrophil degranulation was assessed by elastase, myeloperoxidase (MPO), and alkaline phosphatase (ALP) release as described previously [12]. Nitric oxide and superoxide production were measured with the method used for assessment of macrophage function.

### Statistical analysis

The examined values were expressed as the mean (SD) and compared using the analysis of variance (ANOVA) and Student’s t-test with the STATISTICA PL Software (StatSoft, Poland) and the differences were considered as significant at* p* < 0.05.

## Results

### PRP parameters and contents

Haematological values in all examined rabbits were within the normal range. After the PRP preparation the leukocyte and platelet counts increased significantly (*p* < 0.01) compared to values obtained in whole blood (4.6-fold and 5.5-fold, respectively) (Table 1).

**Table 1:** Mean white blood cells (WBC), red blood cells (RBC), platelets (PLT) concentration in whole blood, platelet rich plasma (PRP) and platelet poor plasma (PPP)

Blood cells/yield	Whole blood	PRP	PPP
WBC (×10^9^/l)	9.05 ± 2.88	41.25 ± 18*	0.1 ± 0.05*
RBC (×10^12^/l)	6.36 ± 0.1	0.82 ± 0.86*	0.4 ± 0.04*
PLT (×10^9^/l)	213.5 ± 24.7	1163.5 ± 420*	8.00 ± 1.73*
Yield (%)	–	537.13 ± 134.8	–

* *p* < 0.01 in comparison with values obtained in whole blood

The contents of the obtained preparations of PRP and AMP were assessed by the MALDI-TOF MS analysis and the molecular weights of peptides were analyzed by the ExPASy MW/pI tool (http://www.expasy.ch/tools/pi_tool.htlm) on the basis of UniProt database (www.uniprot.org).

**Fig. 1:**
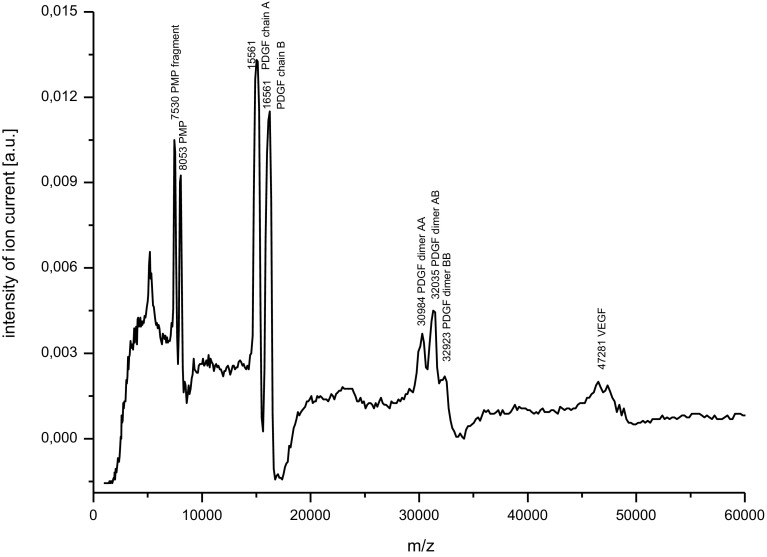
The MALDI TOF MS mass spectrum of components of platelet-rich plasma (PRP) obtained from rabbit blood in the mass range 0–60 000 Da (a.u.—arbitrary units, m—mass of an ion, z—charge of an ion)

In PRP preparation platelet microbicidal peptide (PMP) of the molecular mass of 8053 Da and its fragment of 7530 Da were visible. Apart from antimicrobial platelet peptides also growth factors were detected as Platelet Derived Growth Factor (PDGF) chains A (Isoform A3-accession number P34007), B and dimers AA, AB, and BB of the molecular masses of 15,561, 16,561, 30,984, 32,035, 32,923 Da, respectively. The peak of 47,281 Da reflects Vascular Endothelial Growth Factor (VEGF) (Fig. 1).

### AMP extract contents

**Fig. 2:**
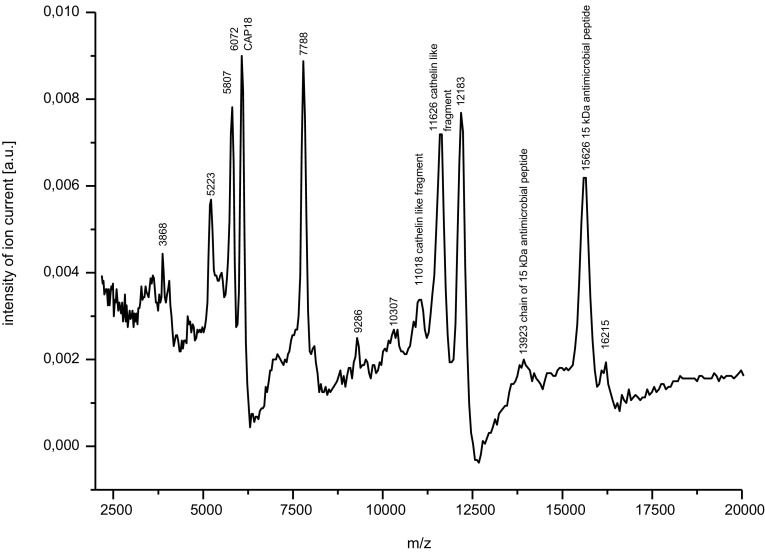
The MALDI TOF MS mass spectrum of rabbit neutrophil extract (fraction 1) in the mass range 2500–17500 Da

In fraction 1 of the neutrophil extract we detected rabbit cathelicidins of higher molecular masses, namely 15,000 Da antimicrobial peptide (as the mass of 15,626 Da) (accession number P26203), 13,923 Da as the chain of 15,000 Da antimicrobial peptide, cathelin-like fragments i.e. two peaks about 11,000 Da, and some peptides of the molecular masses between 6072 and 12,183 Da as the products of fragmentation of CAP 18. Peaks of 3868 and 5223 Da were the fragments of peptides of higher molecular sizes (Fig. 2).

**Fig. 3:**
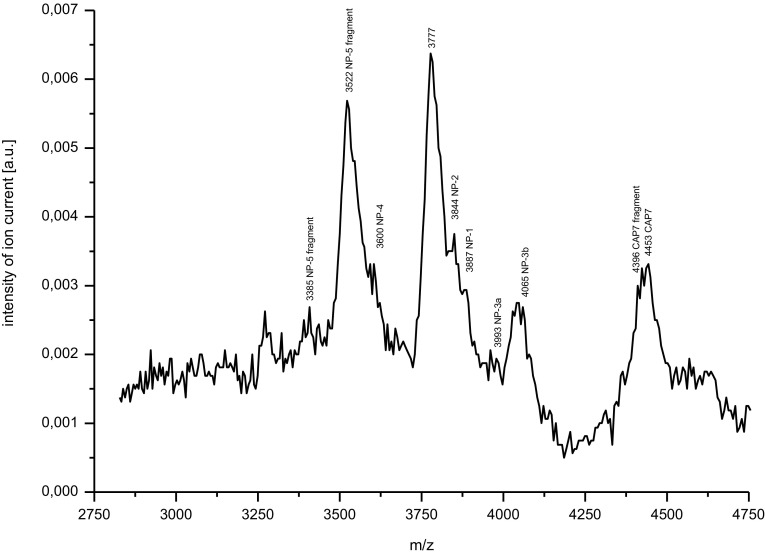
The MALDI TOF MS mass spectrum of fraction 2 contained peptides with low molecular masses (2750–4750 Da) from rabbit neutrophils

In the fraction of low molecular masses (fraction 2) defensins from rabbit neutrophils with the following molecular masses; NP-1 (3887 Da) (accession number P01376), NP-2 (3844 Da) (accession number P01377), NP-3a (3993 Da) (accession number P07469), NP-3b (4065 Da) (accession number P07468), NP-4 (3600 Da) (accession number P07467) and NP-5 (as fragments of 3385 Da and 3522 Da) (accession number P07466) and the fragment of higher defensin NP-1 or NP-3b (3777 Da) were found. Moreover, the antibacterial peptide CAP 7 (accession number P25230) with the molecular size of 4453 Da and its fragment of 4396 Da were detected (Fig. 3).

### Determination of the Mfs function and morphological changes in response to stimulators

**Fig. 4:**
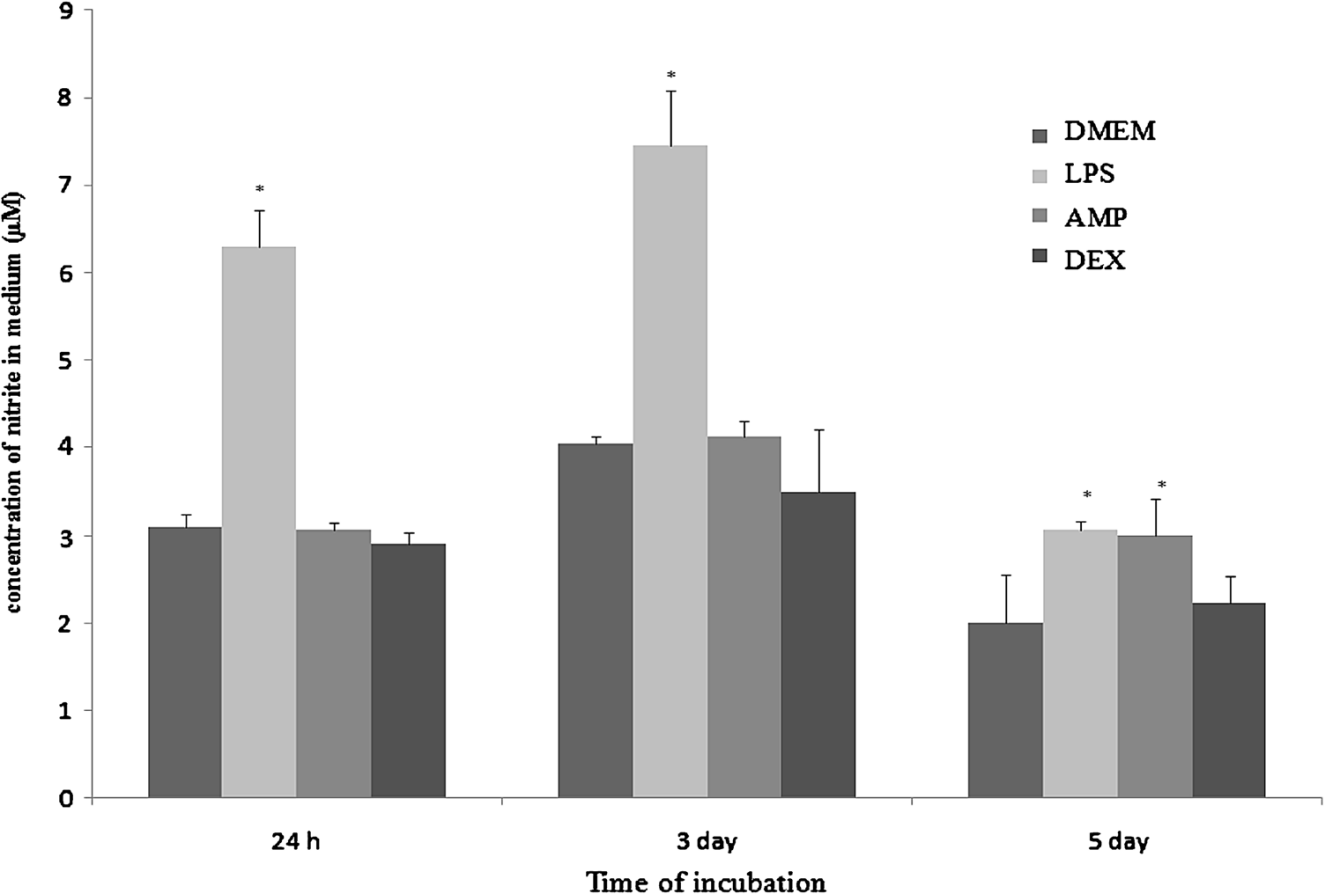
The generation of nitric oxide by cultures of macrophages: unstimulated (described as DMEM), stimulated by LPS (LPS), stimulated by AMP neutrophil extract (AMP) and stimulated by dexamethasone (DEX), after 24 h, 3 days and 5 days of incubation at 37 °C and 5% CO_2_. *p*-values calculated for LPS, AMP, and DEX groups were compared with (Student t-test) to unstimulated cultures (DMEM) and were <0.05. The data are presented as the mean ± SE from six rabbits in duplicate experiment

In cultures stimulated with LPS significant (*p* < 0.05) increase of NO generation was observed in all three time points. In Mfs under the influence of AMP extract in first two measurements the generation of NO was similar to control (DMEM) group. In the 3rd measurement cultures stimulated with AMP extract exert increased response in comparison with DMEM cultures (Fig. 4).

**Fig. 5:**
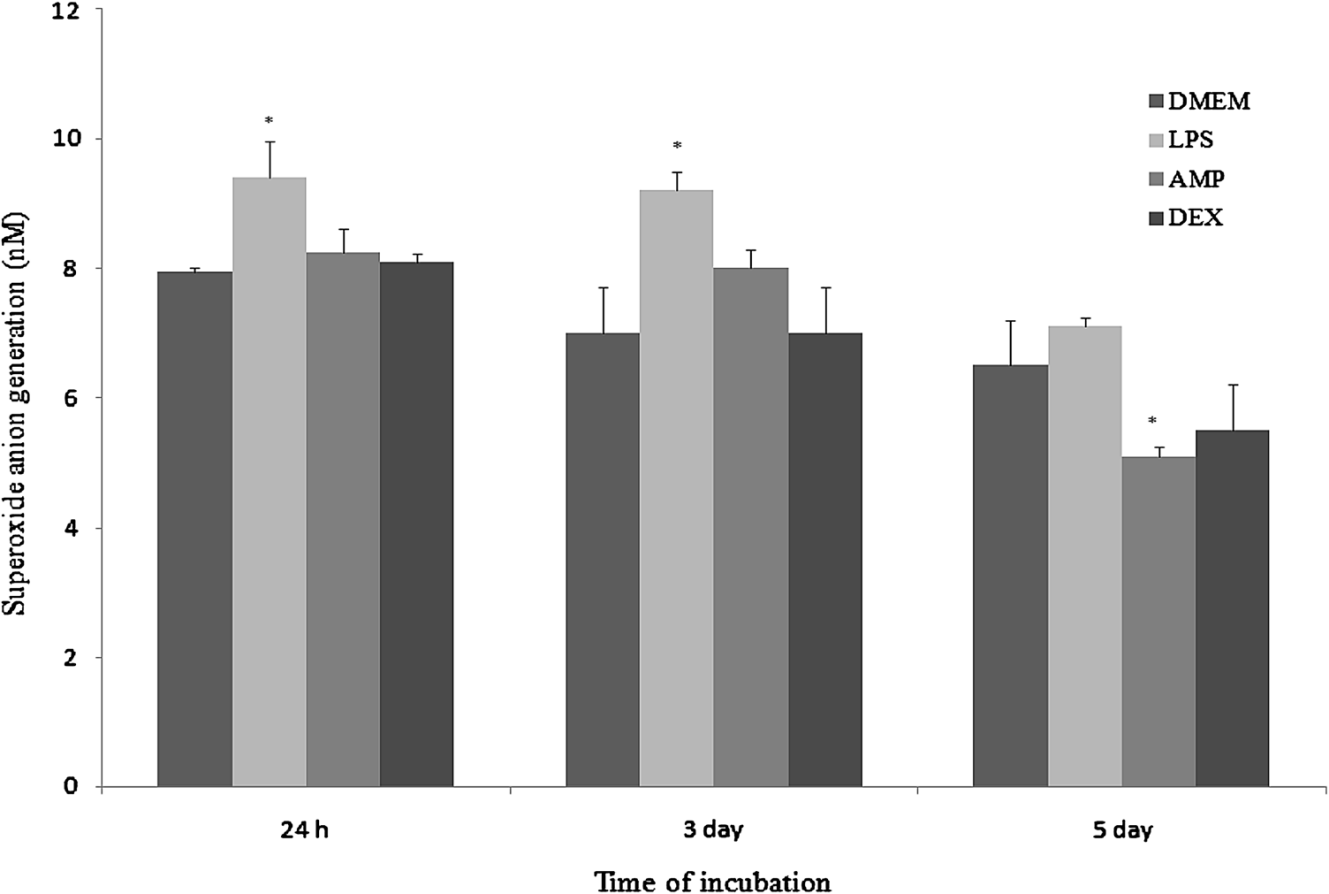
The generation of superoxide anion by cultures of macrophages: unstimulated (described as DMEM), stimulated by LPS (LPS), stimulated by AMP neutrophil extract (AMP) and stimulated by dexamethasone (DEX), after 24 h, 3 days and 5 days of incubation at 37 °C and 5% CO_2_. *P*-values calculated for LPS, AMP, and DEX groups were compared with (Student t-test) to unstimulated cultures (DMEM) and were <0.05. The data shown are the mean of six rabbits ±SE in duplicate experiment

The generation of superoxide under the influence of LPS increased significantly in the first and second measurement (*p* < 0.05), whereas the increase in the third measurement was insignificant. Under the influence of AMP superoxide anion production markedly decreased in comparison with unstimulated culture (Fig. 5).

**Fig. 6:**
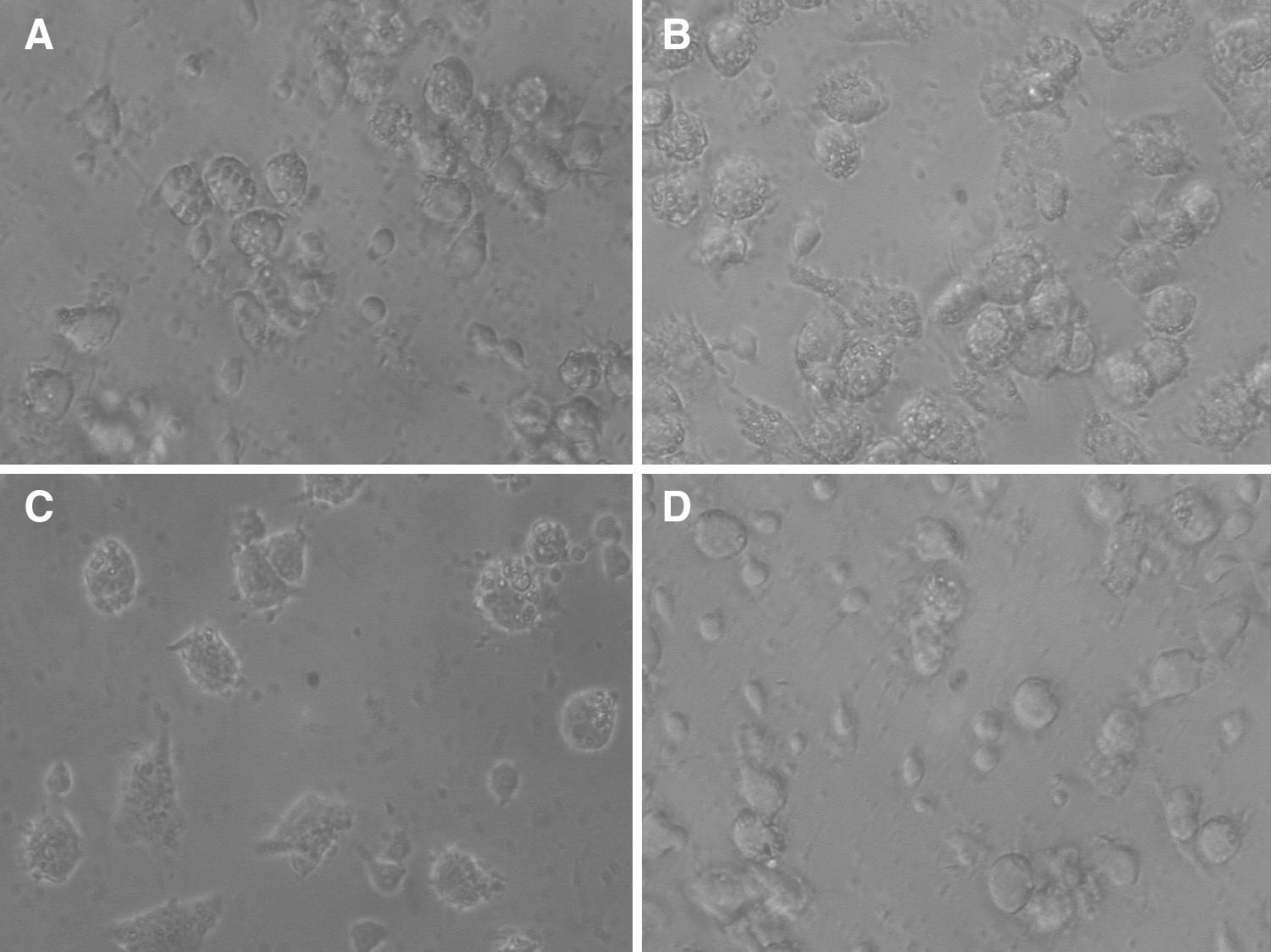
Representative photomicrographs showing untreated **A** and 24 h LPS, AMP and DEX treated monocyte-derived macrophages signed as **B**, **C**, and **D**, respectively. Morphology of untreated **A**, LPS treated **B**, AMP treated **C** and DEX treated **D** macrophages was assessed by phase-contrast microscopy (×40 objective)

**Fig. 7:**
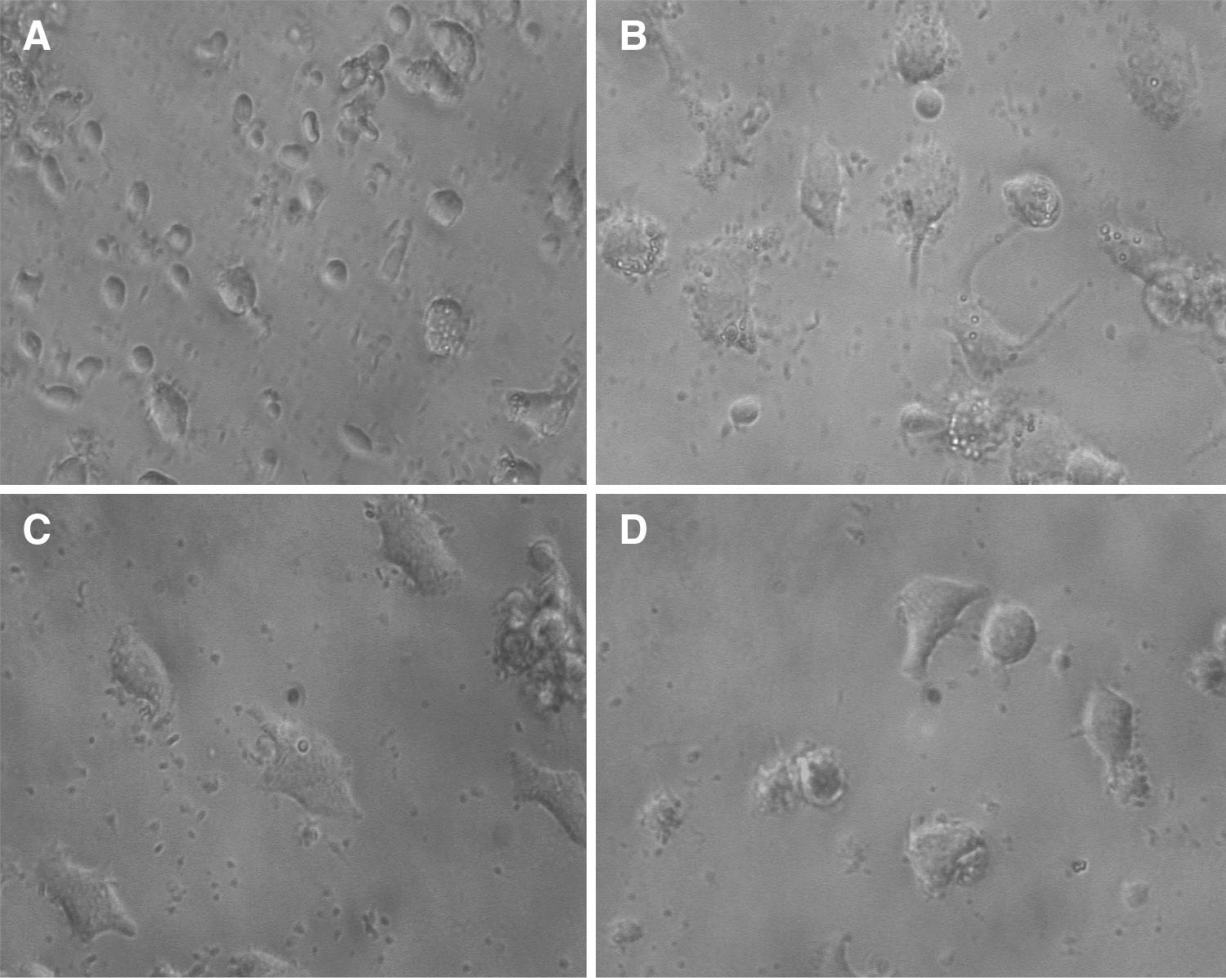
The effect of LPS, AMP and DEX on macrophage phenotype. Adherent peripheral blood-derived macrophages cultured for 3 days in the presence or absence of stimulators. Morphology of untreated **A**, LPS treated **B**, AMP treated **C** and DEX treated **D** macrophages was assessed by phase-contrast microscopy (×40 objective)

Under the standard culture conditions (DMEM) Mfs were heterogenous population of cells with different morphologies, mainly rounded, less spread cells, and occasional dendric-like cells (Fig. 6A). Addition of LPS caused dendric-like morphology with large filopodia within 24 h of stimulation (Fig. 6B). The addition of AMP extract generate dendric like cells with filopodia and some rounded cells (Fig. 6C). In contrast, addition of dexametasone resulted in increase of rounded cells and reduction of large multinucleated cells and spread macrophages (Fig. 6D). After 3 days incubation differences are more clear. Under the influence of LPS in cultures of Mfs dendric-like appearance with long filopodia predominate (Fig. 7B) in comparison with unstimulated (DMEM) culture (Fig. 7A). In culture of AMP extract, in turn, the dendric- like cells with short filopodia predominate (Fig. 7C), whereas DEX causes elongation of cells (Fig. 7D).

### Neutrophil activity in response to stimulators

**Fig. 8:**
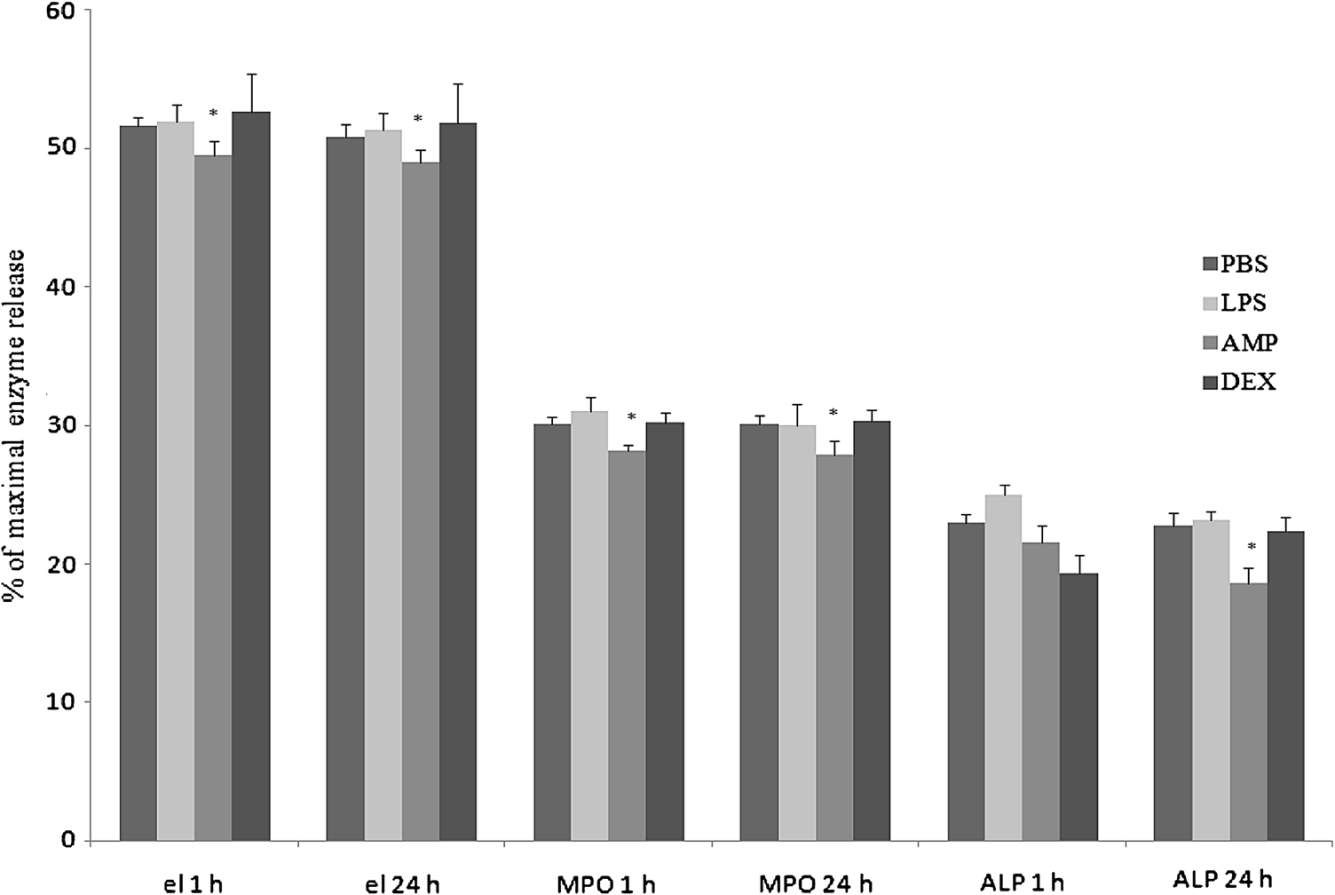
The effect of PBS, LPS, AMP or DEX on degranulation of neutrophils after 1 h and 24 h of culture (el means elastase release, MPO means myeloperoxidase release, ALP means alkaline phosphatase release). Each bar represents the mean of six rabbits in duplicate measurements and error bars represent the ±SE from the mean. P-values calculated for LPS, AMP, and DEX groups were compared with (Student t-test) to unstimulated cultures (PBS) and were <0.05. The data are representative of six rabbits in duplicate experiment

In our experiment addition of LPS (1 µg/ml) to neutrophil cultures did not result in significant increase of degranulation, as estimated on the basis of elastase, MPO, and ALP release, both after 1 h and 24 h of incubation. Under the influence of AMP extract neutrophil elastase release was inhibited, as well as MPO response and ALP release in both measurements after 1 h and after 24 h in comparison with unstimulated culture. The response to DEX was not significant and diverse (Fig. 8).

**Fig. 9:**
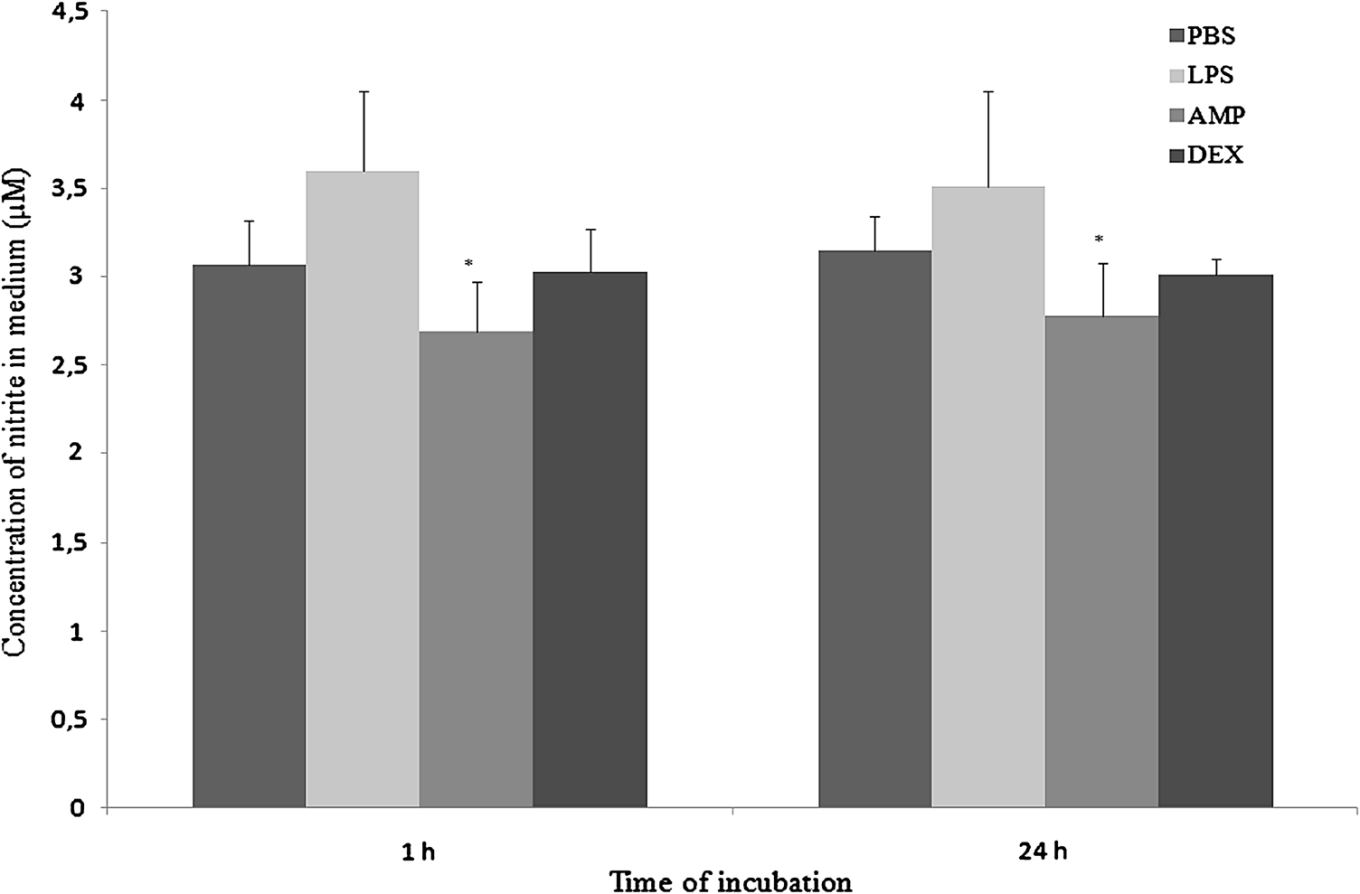
The effect of PBS, LPS, AMP or DEX on generation of nitric oxide by neutrophils after 1 h and 24 h of culture. Each bar represents the mean of six rabbits in duplicate measurements and error bars represent the ±SE from the mean. P-values calculated for LPS, AMP, and DEX groups were compared with Student t-test to unstimulated cultures (PBS) and were <0.05. The data are representative of six rabbits in duplicate experiment

**Fig. 10:**
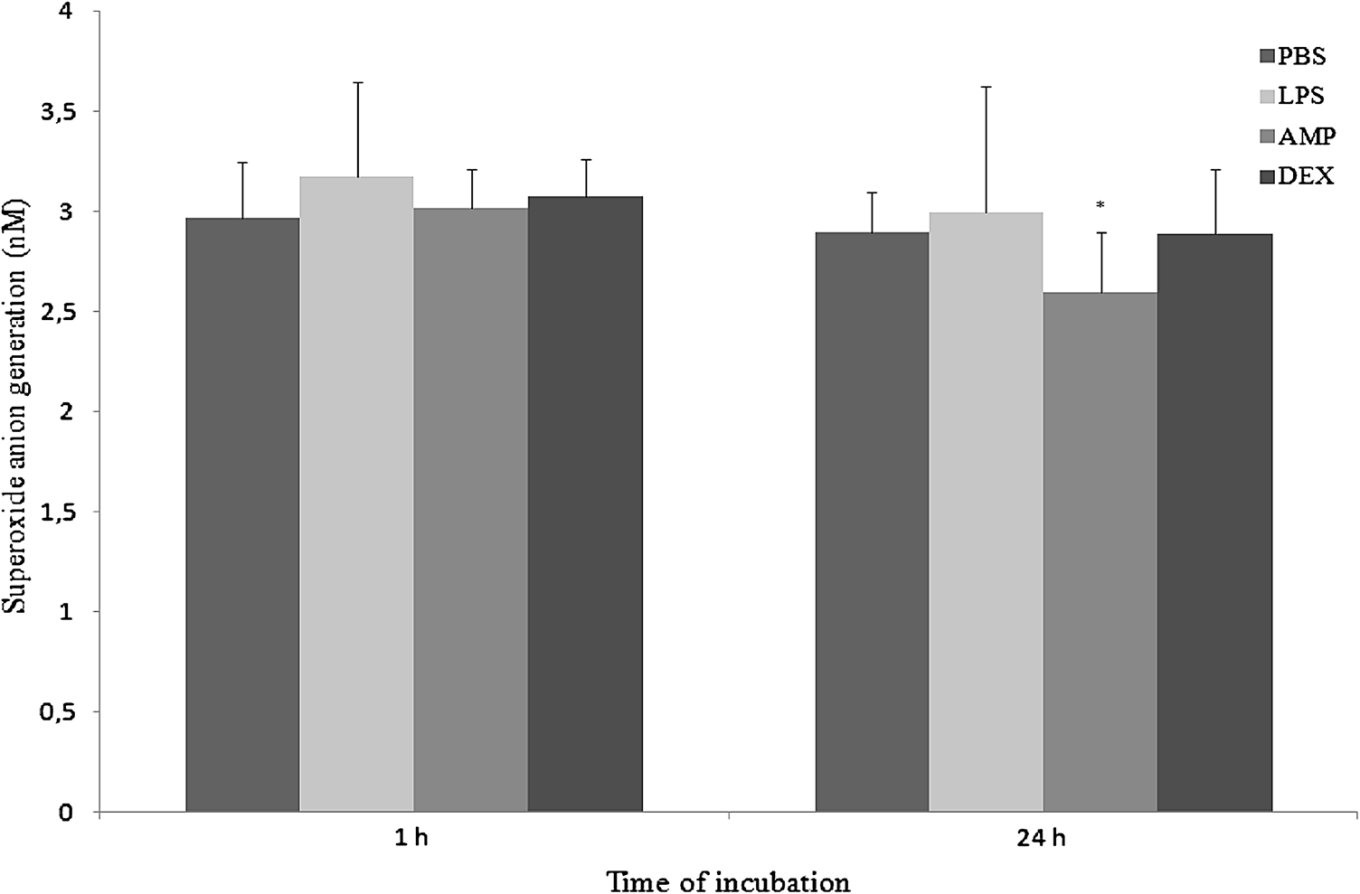
The effect of PBS, LPS, AMP or DEX on generation of superoxide anion by neutrophils after 1 h and 24 h of culture. Each bar represents the mean of six rabbits in duplicate measurements and error bars represent the ±SE from the mean. P-values calculated for LPS, AMP, and DEX groups were compared with Student t-test to unstimulated cultures (PBS) and were <0.05. The data are representative of six rabbits in duplicate experiment

AMP extract significantly (*p* < 0.05) inhibited generation of NO in both measurements, whereas superoxide production increased insignificantly in 1st measurement and decreased significantly (*p* < 0.05) in 2nd measurement. DEX did not influence on respiratory burst of neutrophils (Figs. 9, 10, respectively).

## Discussion

We obtained significant increase (*p* < 0.01) of PLT number in PRP from rabbit blood, (about 5.5-fold). The yield of PLT from whole blood varied between the authors and depended on species and method of separation used [14]. In the study of Jia [15] the 6.9-fold increase in platelets was noted, the other authors achieved 6.4-fold augmentation of PLT [16] and 7.2-fold [17].

We obtained PRP rich in PDGF AB, PDGF BB, PDGF AB, VEGF, and platelet microbicidal peptide. In previous reports for assessment of growth factors, the ELISA tests were applied [18, 19]. We used the MALDI TOF analysis for detection of constituents of PRP because of high sensitivity and reliability of the method.

Our results showed an average increase of 4.6-fold of leukocytes from whole blood of rabbits. Other authors described 5.9-fold increase Jia et al. [15] 3.12-fold [16], 5.0-fold [4] and 2.42 to 5.37-fold [17] increase of WBC dependently of the system used. The role of WBC especially neutrophils as components of PRP still remains controversial. Neutrophils are responsible for the phagocytosis and elimination of foreign pathogens [15], however, WBC release matrix metaloproteinases and produce ROS that can lead to the increased tissue damage. Taking into account these limitations, we considered usage of AMP neutrophil extract for delivery of antimicrobial components without side effects connected with enrichment of PRP with intact WBC.

As estimated, local delivery of AMP increases the antibiotic concentration and simultaneously minimalizes their systemic toxicity [4]. Till now antimicrobial peptides, on the basis of human lactoferrin (hLF-11), have been evaluated in the treatment of osteomyelitis on rabbits models [10].

We estimated that under the influence of LPS Mfs shown increased generation of NO as well as superoxide anion. LPS is capable of polarizing Mfs toward classical proinflammatory activation stage and release reactive species and inflammatory cytokines to fight pathogens [18]. Exposure to LPS *in vitro* causes a drift of inflammatory macrophages into the fully activated stage with an increase in secretion of reactive intermediates, especially nitric oxide [19].

The generation of NO by Mfs increased under the influence of AMP extract in third measurement. On the other hand, the addition of AMP extract to Mfs culture caused inhibition of superoxide anion generation on 5th day of culture, whereas in two first measurements superoxide anion augmented insignificantly. According to Agier [5] human cathelicidin LL-37 significantly decreases NO stimulated by LPS and slightly decrease ROS synthesis. In turn, as estimated by Zughaier et al. [6] AMP enhance respiratory burst in human and murine leukocytes. The influence of neutrophil extract on respiratory burst of rabbit macrophages has not been determined previously.

Macrophages in cultures treated with LPS were dendric-like with large filopodia similar to those described in the report of Ploeger et al. [20]. The influence of AMP extract generate morphological changes in the form of dendric like cells with filopodia and some rounded cells. Does et al. [8] observed changes in shape of human macrophages under the influence of human cathelicidin LL-37, namely appearance of fried egg-shaped macrophages with typical morphology of M1 macrophages. In our experiment Mfs under the influence of dexamethasone revealed the higher proportion of rounded cells, the absence of large multinucleated cell populations and reduced numbers of spread macrophages similar to these in the study of Giles et al. [21]. All cited reports described morphological changes in human macrophages, whereas the knowledge on rabbit macrophages differentiation is very restricted.

We did not observe increased release of elastase, MPO and ALP from neutrophils under the influence of LPS. According to Naegelen et al. [22] *in vitro* stimulation of neutrophils with LPS did not activate degranulation of neutrophils, as estimated on the basis of a lack of mobilization of appropriate granule markers.

AMP extract inhibited degranulation of neutrophils and causes decrease of NO and superoxide anion production. We estimated previously that crude porcine extract increased activity of rabbit neutrophils [12]. This effect might be evoked by different contents of this preparation and its heterogeneous source. Some data indicated that cathelicidins directly activate neutrophils and LL-37 stimulates generation of ROS in process mediated by a flavoenzyme and via increase in intracellular Ca^2+^ concentration [5]. Contrary, as shown in the study of Alalwani et al. [23] cathelicidin modulates neutrophil functions by suppressing the release of proinflammatory mediators and increasing antimicrobial activity.

In conclusion, the assessment of PRP and AMP extract contents was done using the MALDI TOF method. The addition of the AMP extract into cells cultures decreased superoxide in Mfs and inhibited degranulation and respiratory burst in neutrophils, thus diminished proinflammatory activity of both studied populations of WBC, preventing excessive inflammation. Based on these findings AMP neutrophil extract could be used for improvement of antimicrobial and healing activities of PRP. These features of AMP will enrich the therapeutic values of PRP, which is widely used in clinical practice.
